# Time-of-Day-Dependent Effects of Aerobic Exercise on Carotid Hemodynamics in Sedentary Adults

**DOI:** 10.3390/biology14060713

**Published:** 2025-06-17

**Authors:** Bingyi Shen, Haibin Liu, Shuying Zhang, Lihong Chen, Guangrui Yang

**Affiliations:** 1 School of Bioengineering, Dalian University of Technology, Dalian 116024, China; bingyi@mail.dlut.edu.cn; 2 School of Clinical Medicine, Shanghai University of Medicine & Health Sciences, Shanghai 201318, China; zhangsy_23@sumhs.edu.cn; 3 School of Sport and Health Sciences, Dalian University of Technology, Dalian 116024, China; liuhaibin@dlut.edu.cn; 4 Health Science Center, East China Normal University, Shanghai 200241, China

**Keywords:** aerobic exercise, exercise timing, hemodynamics, vascular health, circadian rhythm

## Abstract

Aerobic exercise (AE) modulates vascular function through hemodynamic responses, thereby influencing cardiovascular health and risk, with the circadian rhythm system playing a crucial role. This chronobiological study investigated diurnal variations in exercise-induced hemodynamic changes in the common carotid artery. In a randomized crossover trial, twenty-two sedentary adults completed eight AE interventions (one per laboratory visit day), with each session performed at one of eight evenly distributed time points (from 06:00 to 20:00). Vascular ultrasound imaging and hemodynamic parameter calculations were performed both pre- and post-exercise. Compared to other time points, AE at 06:00 and 18:00 induced a greater and more sustained increase in mean flow rate and wall shear stress (WSS). Moreover, AE at 06:00 was associated with a smaller increase in oscillatory shear index and a larger decrease in peripheral resistance compared to other time points. Exercise-induced hemodynamic responses exhibited significant temporal variations. These findings emphasize the importance of exercise timing in optimizing vascular benefits for sedentary individuals.

## Simple Summary

Biological rhythms strongly influence blood vessel health, but it remains unclear how the timing of exercise affects blood flow in key arteries like the carotid artery, which supplies the brain. This study uniquely examines how scheduling aerobic exercise across eight evenly spaced intervals (from 06:00 to 20:00) modulates hemodynamic responses. High-resolution temporal analysis revealed that exercise at 06:00 improved shear stress patterns, vascular regulation, and blood supply to the brain. Exercise at 18:00 also showed positive effects. By identifying these beneficial time windows, this research helps design practical, science-based exercise schedules—particularly for sedentary individuals with limited time for exercise—to optimize vascular health benefits.

## 1. Introduction

Cardiovascular diseases (CVDs) are a leading cause of mortality worldwide, driven by risk factors such as physical inactivity and hypertension [1]. Observing the fluctuations of blood pressure (BP), vascular tone, and endothelial function throughout the day reveals the existence of circadian rhythms in the cardiovascular system [2]. For instance, BP rapidly rises in the morning and decreases by 10–20% during nighttime sleep, with the morning BP surge potentially explaining the higher likelihood of CV events occurring during this period [3,4]. These circadian rhythms are regulated by the master biological clock located in the suprachiasmatic nucleus (SCN) of the hypothalamus as well as peripheral clocks in CV tissues, which are influenced by external factors such as light, diet, and exercise [5,6,7].

Exercise is a key non-pharmacological strategy for improving CV health, and its effectiveness may be influenced by the timing of exercise [8,9,10]. For instance, exercise at 16:00 decreases both BP and peripheral resistance (PR), compared to a slight increase after 8:00 exercise [11]. A prospective cohort study suggested that physical activity in the late morning lowers the CV risk more than midday exercise [12]. However, a recent study found that evening exercise may reduce CVDs and mortality in obese adults [10]. Current research limitations include narrow temporal sampling windows and insufficient characterization of key hemodynamic parameters. Of particular clinical relevance, common carotid artery (CCA) hemodynamics serve as critical determinants of cerebrovascular perfusion and systemic vascular health [13,14]. Although exercise intensity and mode affect CCA stiffness and hemodynamics [15,16,17], the chronobiological dimension remains underexplored.

Previous studies have explored the effects of various exercise patterns—duration, intensity, frequency, and type—on vascular health [18,19,20]. Adults are recommended to engage in more than 150 min of moderate-intensity (each session lasting over 30 min), 75 min of vigorous-intensity (each session lasting over 20 min) aerobic exercise (AE) per week, or an equivalent combination [21,22]. AE, characterized by the dynamic movement of large muscles over a period, promotes vascular remodeling and enhances endothelial function [23,24]. Despite established benefits, the temporal optimization of AE regimens remains complex and understudied, particularly regarding hemodynamic outcomes in sedentary populations. This study employs a high-resolution chronobiological approach to map CCA hemodynamic responses to AE administered at eight distinct circadian time points, aiming to provide valuable insights for designing personalized exercise regimens for sedentary adults.

## 2. Materials and Methods

### 2.1. Subjects

This randomized crossover trial was conducted at Dalian University of Technology, China. From an initial pool of 107 candidates, 24 sedentary male participants meeting the inclusion criteria were ultimately enrolled. Two participants withdrew from the experiment due to scheduling conflicts, and data from 22 subjects were ultimately included in the analysis. They were identified through the International Physical Activity Questionnaire (IPAQ-SF) assessment as physically inactive (they did not meet the World Health Organization’s recommendation for adults) and had more than eight hours of sedentary behavior per day in the past three months [21]. They were non-obese (BMI 18.5–23.9 kg/m^2^), non-smokers, without sleep disorders (Pittsburgh Sleep Quality Index score ≤ 5) [25,26], and had no history of cardiovascular or metabolic diseases.

### 2.2. Experimental Design

**Figure 1:**
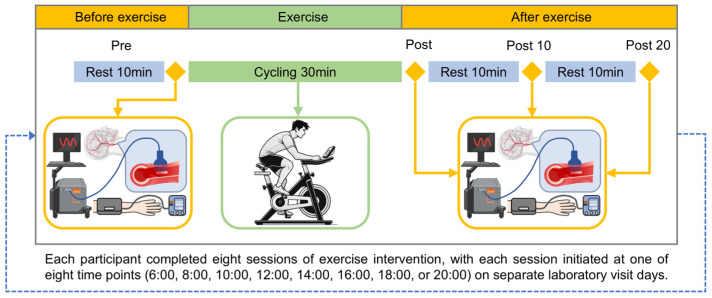
The experimental protocol for each aerobic exercise (AE) session. Each participant completed eight exercise interventions that started at different times (06, 08, 10, 12, 14, 16, 18, and 20 AE) and were separated by at least 60 h from the previous one.

At the first visit to the laboratory before the AE intervention, body composition measurements (MC-980MA, Tanita, Tokyo, Japan) were taken (Table 1). Before the exercise intervention, each participant was required to wear an accelerometer (ActiGraph GT9X Link, ActiGraph, Pensacola, FL, USA) and keep a sleep diary for three consecutive days to monitor their habitual sleep patterns. The same monitoring is implemented during the exercise intervention period. Each participant underwent 8 sessions of exercise interventions, with different start times scheduled at 6:00, 8:00, 10:00, 12:00, 14:00, 16:00, 18:00, and 20:00 (06, 08, 10, 12, 14, 16, 18, and 20 AE). According to the experimental flow for each session (Figure 1), they rested quietly for at least 10 min before baseline testing. The ultrasound image acquisition was performed before (Pre) and immediately after exercise (Post), as well as at 10 min (Post10) and 20 min (Post20) post-exercise. Our previous study investigated hemodynamics after exercise and found that some parameters had already recovered by 20 min, with nearly all parameters returning to baseline by 30 min [16]. To ensure the results were not mutually influenced, the order of interventions was randomized, and a washout period of at least 60 h was allowed between interventions [27]. Participants were instructed to avoid vigorous physical activity, alcohol, drugs, and caffeine intake in the 24 h preceding the experiment.

The AE intervention involved continuous cycling for 30 min using a power bike (Powermax-Ⅷ, Combi Wellness, Tokyo, Japan). Participants’ target heart rate range was calculated based on their reserve heart rate (HRR) obtained through the Bruce protocol exercise test (MetaLyzer 3B—R2, Cortex, Southampton, UK) [28,29]. The exercise intensity was set at 60–70% HRR. The power bike load was set at 3 kp, and participants warmed up for 5 min before exercise intervention to elevate their HR to the target range. During exercise, participants wore a wireless sensor (Polar team pro, Polar, Kempele, Finland) on their chest to monitor HR in real time. This allowed for timely speed adjustments to ensure that the exercise intensity met the required level.

### 2.3. Measurements and Calculations of Local Hemodynamics

The diameter and center-line flow velocity (FV) waveforms of the right common carotid artery were measured using the color Doppler ultrasound (Prosound Alpha 7, Aloka, Tokyo, Japan), with the measurement and calculation methods detailed in our previous studies [15,16]. Simultaneously, an electronic sphygmomanometer (HEM-7136, Omron, Kyoto, Japan) was used to record BP on the left arm. MATLAB (Version 9.14.0.2206163 (R2023a), The MathWorks, Inc., Natick, MA, USA) was utilized for programming to extract the diameter and FV waveforms and synchronize them with heart rate signals (beat-to-beat recording). The diameter waveforms and BP are then used to obtain the corrected BP waveform of the carotid artery. The extracted data are processed using the following formulas to obtain the hemodynamic parameters discussed in this paper:

Blood Pressure (*P*)

(1) $$P_{i}=\frac{P_{s\_mean}-P_{d\_mean}}{D_{max}-D_{min}}\times \left(D_{i}-D_{min}\right)+P_{d\_mean}$$

(2) $$p_{m}=p_{d}+\frac{1}{3}(p_{s}-p_{d})$$

where *P_s_mean_* and *P_d_mean_* are the systolic and diastolic pressures (SBP and DBP) of the brachial artery; *D*_max_ and *D*_min_ are the max and min values of *D*; and *D_i_* and *P_i_* are the *D* and the calculated values of BP at the same time. The maximum and minimum values of *P_i_* are the calibrated SBP (*P_s_*) and DBP (*P_d_*), respectively. The mean arterial pressure (*P_m_*) was calculated by formula with a form factor equal to 33% [30,31].

Flow Rate (*Q*)

(3) $$Q=2\pi {\bar{R_{0}}}^{2}\int_{0}^{1}y\cdot u(y,t)dy$$

where *R*_0_ is the average of the radius over time during a cardiac cycle; *t* is the period of one cardiac cycle; and *y* = *r*/*R*_0_, in which *r* is the radial coordinate. Moreover, *u*(*y*, *t*) satisfies the following expression:

(4) $$u(y,t)=\sum_{n=-\infty }^{+\infty }\frac{J_{0}(\alpha_{n}j^{\frac{3}{2}})-J_{0}(\alpha_{n}j^{\frac{3}{2}}y)}{J_{0}(\alpha_{n}j^{\frac{3}{2}})-1}u(0,\omega_{n})e^{j\omega_{n}t}$$

(5) $$u(0,t)=\sum_{n=-\infty }^{+\infty }u(0,\omega_{n})e^{j\omega_{n}t}$$

where $J_{0}$ is the zero-order Bessel function of the first kind and $j=\sqrt{-1}$. $\alpha_{n}$ is the Womersley number and n is the harmonic number. $\alpha_{n}=R_{0\;}\sqrt{\rho \omega_{n}/n\;}$, in which *ρ* is the density of blood and *η* is blood viscosity. n = 0.004 Pa·s and *ρ* = 1050 kg/m^3^. $\omega_{n}=2\pi nf$ is the angular frequency and f is the base frequency. u (0, $\omega_{n}$) is the n harmonic component of the flow velocity (*V*). The maximal harmonic number n was computed as 20.

Apparent Elastic Modulus (*E_p_*))

*E_p_* reflects the degree of change in *D* caused by BP.

(6) $$E_{p}=\frac{p_{s}-p_{d}}{R_{s}-R_{d}}\cdot R_{d}$$

Wall Shear Stress (*τ_w_*)

Wall shear stress (WSS), the frictional force exerted by blood flow on the vascular walls, is a hemodynamic indicator associated with atherosclerosis [32].

(7) $$\tau_{w}=\frac{\eta }{\bar{R}}\sum_{n=-\infty }^{+\infty }\frac{\alpha_{n}j^{\frac{3}{2}}J_{1}(\alpha_{n}j^{\frac{3}{2}})}{J_{0}(\alpha_{n}j^{\frac{3}{2}})-1}u(0,\omega_{n})e^{j\omega_{n}t}$$

where $J_{1}$ is the first-order Bessel function of the first kind.

Oscillatory Shear Index (*OSI*)

(8) $$OSI=\frac{1}{2}\left(1-\frac{\left|\int_{0}^{T}\tau_{w}dt\right|}{\int_{0}^{T}\left|\tau_{w}\right|dt}\right)$$

Dynamic Resistance (*DR*)

(9) $$DR=\frac{p_{s}-p_{d}}{Q_{max}-Q_{min}}$$

where *Q*_max_ and *Q*_min_ are the maximum and minimum values of *Q*, respectively.

Peripheral Resistance (*PR*)

(10) $$PR=\frac{p_{m}}{Q_{mean}}$$

where *Q*_mean_ is the mean value of *Q*.

Pulsatility Index (*PI*)

(11) $$PI=\frac{V_{max}-V_{min}}{V_{mean}}$$

where *V*_max_, *V*_min_, and *V*_mean_ are the maximum, minimum, and mean values of *V*, respectively.

### 2.4. Data Processing and Statistical Analysis

Using G Power (Version 3.1.9.7, Heinrich Heine University Düsseldorf, Düsseldorf, Germany), a repeated measures ANOVA with within factors determined that at least 11 participants are required to achieve a medium effect size (0.30) with a power of 0.80 and an α level of 0.05. All data were presented as the mean ± SE. Due to the variability of baseline across different times of the day for most hemodynamic parameters, post-exercise results are presented not only as absolute quantities (Pre, Post, Post10, and Post20 refer to the results before exercise, immediately after exercise, and 10 min and 20 min after exercise, respectively) but also as baseline-adjusted changes (ΔPost = Post − Pre; ΔPost10 = Post10 − Pre; ΔPost20 = Post20 − Pre), representing the deviation from pre-exercise at each post-exercise time point. ANOVA was used to analyze the effects of different time points on hemodynamic parameters. The level of statistical significance was set as *p* < 0.05.

## 3. Results

### 3.1. Effects of Timed AE on Blood Pressure (BP) and Arterial Elasticity

**Figure 2:**
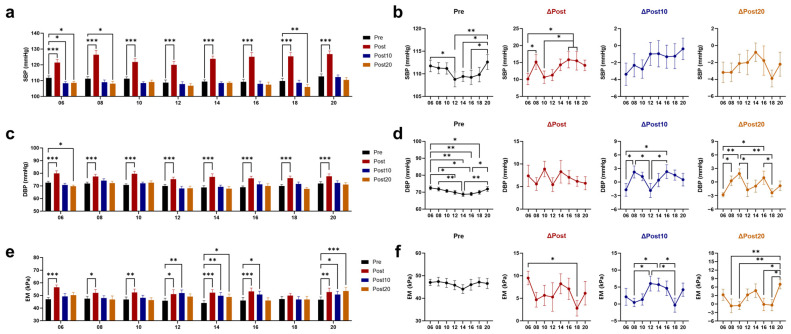
Effects of AE on blood pressure and arterial elasticity at different time points. Response of systolic blood pressure (SBP) (**a**) and its post-exercise changes (**b**) to chrono-exercise; Response of diastolic blood pressure (DBP) (**c**) and its post-exercise changes (**d**) to chrono-exercise; Response of apparent elastic modulus (EM) (**e**) and its post-exercise changes (**f**) to chrono-exercise. * *p* < 0.05, ** *p* < 0.01, *** *p* < 0.001.

The resting values of systolic blood pressure (SBP) and diastolic blood pressure (DBP) fluctuated throughout the day, with a higher level before 06 and 20 AE. Immediately after exercise, both SBP and DBP increased, with the increase in SBP being smaller after 06 and 10 AE than that after 16 and 18 AE. At 10 min after 06 AE, the SBP was significantly lower than pre-exercise, and the DBP showed a more pronounced decreasing trend compared to 08 and 16 AE. At 20 min post-exercise, SBP was observed to be lower than baseline after 06, 08, and 18 AE. DBP was only lower than baseline after 06 AE, with a greater decrease compared to 08, 10, and 16 AE (Figure 2a–d). Apparent elastic modulus (EM) was used to measure arterial elasticity, with higher values indicating greater stiffness and poorer elasticity. After 08 and 18 AE, EM tended to increase less post-exercise and showed greater recovery at 10 min and 20 min post-exercise. Only the 14 and 20 AE failed to recover EM at 20 min post-exercise, with the most significant increase observed after 20 AE (Figure 2e,f).

### 3.2. Effects of Timed AE on Cerebral Blood Supply

**Figure 3:**
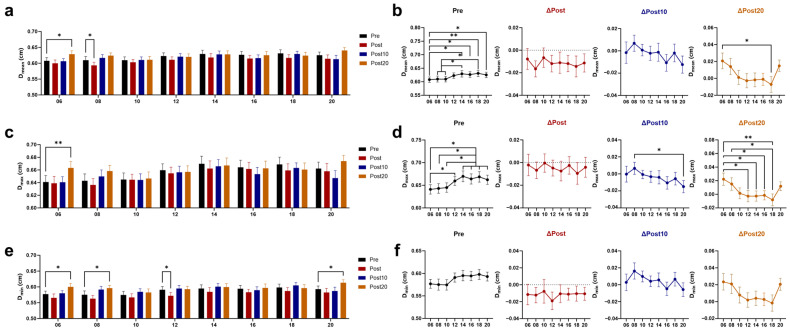
Effects of AE on diameter at different time points. Response of the mean values of diameter (D_mean_) (**a**) and its post-exercise changes (**b**) to chrono-exercise; Response of the maximum values of diameter (D_max_) (**c**) and its post-exercise changes (**d**) to chrono-exercise; Response of the minimum values of diameter (D_min_) (**e**) and its post-exercise changes (**f**) to chrono-exercise. * *p* < 0.05, ** *p* < 0.01.

**Figure 4:**
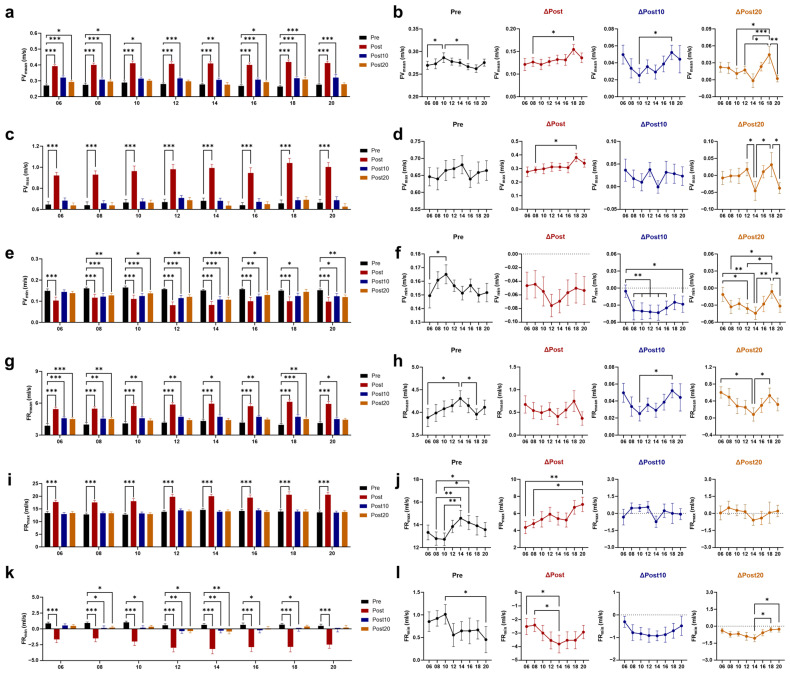
Effects of AE on blood supply at different time points. Response of the mean values of center-line flow velocity (FV_mean_) (**a**) and its post-exercise changes (**b**) to chrono-exercise; Response of the maximum values of center-line flow velocity (FV_max_) (**c**) and its post-exercise changes (**d**) to chrono-exercise; Response of the minimum values of center-line flow velocity (FV_min_) (**e**) and its post-exercise changes (**f**) to chrono-exercise; Response of the mean values of flow rate (FR_mean_) (**g**) and its post-exercise changes (**h**) to chrono-exercise; Response of the maximum values of flow rate (FR_max_) (**i**) and its post-exercise changes (**j**) to chrono-exercise; Response of the minimum values of flow rate (FR_min_) (**k**) and its post-exercise changes (**l**) to chrono-exercise; The negative values of FR_min_ represent retrograde FR. * *p* < 0.05, ** *p* < 0.01, *** *p* < 0.001.

The mean (D_mean_) and maximum (D_max_) diameters were smaller before 06 AE and showed higher values at 20 min after 06 AE, with the increase being greater than 18 AE (Figure 3a–d). The baseline and the change after exercise of the minimum value (D_min_) did not exhibit a significant time difference, while the D_min_ at 20 min after 06, 08, and 20 AE were significantly higher than pre-exercise (Figure 3e,f). The mean and maximum values of center-line flow velocity (FV_mean_ and FV_max_) both increased immediately after exercise (Figure 4a–d), while the minimum values (FV_min_) decreased (Figure 4e,f). The changes in flow rate (FR) were similar to those in FV, with both the mean and maximum values (FR_mean_ and FR_max_) increasing immediately (Figure 4g–j). The minimum value (FR_min_) decreased, with a reverse blood flow increase observed (Figure 4k,l). At 20 min post-exercise, FV_mean_ remained significantly higher than baseline after 06, 08, 16, and 18 AE, and FR_mean_ was still not recovered at these time points, except for 16 AE.

The larger increases in FR_mean_ after 06 and 18 AE correspond to the greater increase in D_mean_ after 06 AE and FV_mean_ after 18 AE.

### 3.3. Effects of Timed AE on Wall Shear Stress (WSS) and Vascular Resistance

**Figure 5:**
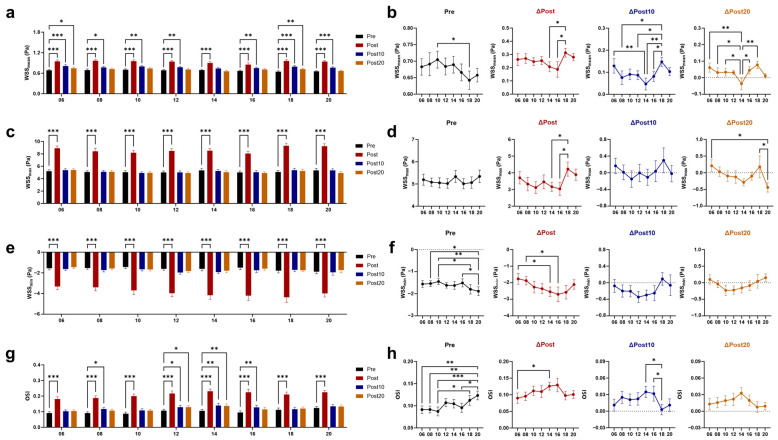
Effects of AE on wall shear stress at different time points. Response of the mean values of wall shear stress (WSS_mean_) (**a**) and its post-exercise changes (**b**) to chrono-exercise; Response of the maximum values of wall shear stress (WSS_max_) (**c**) and its post-exercise changes (**d**) to chrono-exercise; Response of the minimum values of wall shear stress (WSS_min_) (**e**) and its post-exercise changes (**f**) to chrono-exercise; The negative values of WSS_min_ represent retrograde WSS; Response of oscillatory shear index (OSI) (**g**) and its post-exercise changes (**h**) to chrono-exercise. * *p* < 0.05, ** *p* < 0.01, *** *p* < 0.001.

**Figure 6:**
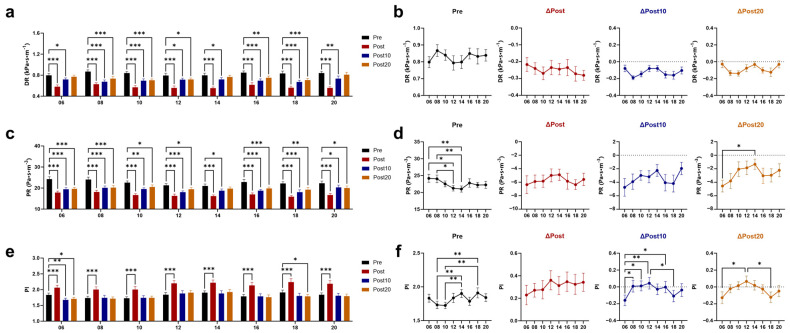
Effects of AE on vascular resistance at different time points. Response of dynamic resistance (DR) (**a**) and its post-exercise changes (**b**) to chrono-exercise; Response of peripheral resistance (PR) (**c**) and its post-exercise changes (**d**) to chrono-exercise; Response of pulsatility index (PI) (**e**) and its post-exercise changes (**f**) to chrono-exercise. * *p* < 0.05, ** *p* < 0.01, *** *p* < 0.001.

Immediately after exercise, the mean (WSS_mean_), maximum (WSS_max_), and minimum (WSS_min_; the negative values of WSS_min_ represent retrograde WSS) values of WSS all increased (Figure 5a–f). The greatest increase in WSS_mean_ and WSS_max_ was shown immediately after 18 AE. Both at 10 and 20 min after 06 and 18 AE, WSS_mean_ remained higher than baseline, with a greater increase compared to 14 AE. The smaller increase in WSS_min_ was observed immediately after 06 and 08 AE, and WSS_min_ had already returned to baseline at 10 min post-exercise. The oscillatory shear index (OSI) reflects the proportion of retrograde WSS to total WSS [33]. Consistent with the changes in WSS_min_, OSI immediately increased after exercise at any time, with the minimal increase observed immediately after 06 AE, and it returned to baseline at 10 min after 06 AE (Figure 5g,h). Immediately and 10 min after exercise, both dynamic resistance (DR) and peripheral resistance (PR) were reduced, with no time difference (Figure 6a–d). At 20 min post-exercise, PR has not fully recovered, with the greatest decrease observed after 06 AE. The pulsatile index (PI) increased immediately after exercise and remained different from the baseline at 20 min after 06 and 18 AE (Figure 6e,f).

## 4. Discussion

This study investigated the effects of timed exercise on vascular health in sedentary adults by performing exercise interventions at eight distinct time points. The research revealed that hemodynamic responses to a single session vary significantly depending on exercise timing. Notably, exercising at 06:00 and 18:00 demonstrated greater health benefits, including improved cerebral blood supply and optimized wall shear stress patterns. This can inform optimal exercise plans to enhance cardiovascular health.

The CCA is crucial in supplying oxygen and nutrients to the brain, with local FV and FR serving as key indicators of cerebral blood supply [34]. Cerebral blood flow changed from morning to night, likely reflecting the circadian rhythm controlled by the SCN [35]. The acute increases in mean and maximum FV and FR post-exercise are potentially due to neurohumoral regulation boosting cardiac output [36]. The effect of exercise at 06:00 and 18:00 on increasing the mean FR lasts longer. This may be explained by the more lasting increases in D at 06:00 and FV at 18:00, which could also indicate a more effective stimulation of the heart’s pumping function. To enhance blood flow, AE is recommended to be performed at 06:00 and 18:00.

The bifurcation area of the CCA is a common site for atherosclerotic plaques [37]. The vessel wall is subjected to vertical (BP) and parallel (WSS) mechanical forces from blood flow, both of which play key roles in arterial stiffness and endothelial function [38,39]. Previous research has delineated two distinct patterns of diurnal blood pressure variation, both critical to understanding vascular dynamics [4,40]. The first pattern, identified by Millar-Craig et al., features a pronounced morning surge in blood pressure followed by a gradual decline throughout the day [40]. This was observed in a study of 20 hypertensive and 5 normotensive individuals, with a single peak occurring in the mid-morning. The second pattern, corroborated by recent studies involving both normotensive and hypertensive populations, exhibits a biphasic rhythm with two peaks—one in the early morning and another in the evening—separated by an afternoon decline [3,41,42]. A salient commonality among these patterns is the morning surge. Observed variations may be attributed to individual differences, group characteristics, or refinements in measurement techniques. Although participants’ average wake-up time advanced by approximately 1 h during early morning exercise, our study recorded elevated resting blood pressure in both morning and evening measurements. This BP pattern aligns with the two daytime peaks, supporting the validity of our approach. Additionally, research indicates that blood pressure reactivity is primarily driven by the endogenous circadian rhythm of the cardiovascular system rather than wake-up time [43,44]. To eliminate confounding effects from baseline fluctuations on post-exercise outcomes, we also assessed the relative changes in vascular parameters using delta values to evaluate the impact of exercise timing. BP increased immediately post-exercise, with more intense rises shown after 08, 16, and 18 AE. During exercise, blood circulation increases to support muscle activity, which induces an acute increase in BP, and repeated exercise leads to arterial adaptation. Given that morning physiological phenomena—including morning BP surges, enhanced sympathetic activity, and increased platelet aggregability—are linked to higher CV events [45,46], although healthy adults can tolerate significant BP fluctuations without adverse symptoms, early vigorous exercise may be inadvisable for those with CV risks. Nonetheless, our study included all daytime time points, including 06:00 and 08:00, to provide a complete chronobiological profile. Excluding any time window, even those potentially less favorable, would have limited the comparative value and completeness of the findings. Post-exercise hypotension (PEH)—a BP drop typically occurring after exercise that lasts minutes up to 24 h—was observed. SBP decreased after 06, 08, and 18 AE, and DBP decreased after 06 AE. The pronounced morning PEH may relate to higher baseline BP levels and circadian regulation, while late afternoon reduction may be due to decreased sympathetic nervous system activity and increased parasympathetic dominance [47,48].

WSS is a hemodynamic indicator associated with atherosclerosis [32]. Low WSS contributes to the formation of aneurysms and atherosclerotic plaques [49,50], while high WSS helps maintain endothelial function [51]. The mean WSS significantly increased after exercise, with the effects lasting longer after 06 and 18 AE. OSI, which reflects fluctuations in WSS, was at a lower level before 06, 08, and 10 AE and showed a smaller acute increase after 06 AE. Sustained WSS elevation improves endothelial function by increasing nitric oxide (NO) bioavailability and reducing reactive oxygen species (ROS), leading to vasodilation [52]. High WSS lowers the expression of pro-inflammatory factors like endothelin-1 (ET-1) [53] and vascular cell adhesion molecule-1 (VCAM-1) [54], while high OSI increases the expression of ET-1, NADPH oxidase (an enzyme that promotes the production of ROS), and VCAM-1 [55,56,57]. The arterial wall’s elastin fibers allow significant dilation during systole, with elastic recoil during diastole ensuring smooth blood flow [58]. The acute decrease in DR and PR, along with the increase in PI, suggests an improvement in vascular compliance. PR was at a higher resting level before 06 and 08 AE, but the greatest decrease was observed after 06 AE, indicating better vasodilation and lower resistance to blood flow. Considering the changes in WSS pattern and PR, AE at 06:00 is recommended for improving endothelial health and vascular regulation.

While this study highlights the impact of exercise timing on hemodynamics, several limitations exist. First, the inclusion of only healthy adult men limits the generalizability of the findings, particularly to populations with CVDs. However, evidence suggests no significant sex differences in core cardiovascular responses (blood pressure, heart rate, stroke volume) when standardized for body composition [59,60,61], implying potential applicability of these findings to female populations. Although no adverse symptoms were reported in healthy adults, future studies should focus on individuals with CVD or those at risk to better understand the effects of exercise timing on CVD management and establish evidence-based safety parameters for morning exercise. Second, while no standardized diet was provided, participants were required to maintain a healthy diet [62]. They followed protocol-defined meal schedules (07:00, 11:00, 17:00) during lab visits and maintained a pre-exercise fasting period of at least 30 min to control for acute dietary confounders. Third, to enhance the ecological validity, the investigation did not enforce wake-up times before 06:00, though this may introduce a confounding effect. Crucially, even when the interval between wake time and exercise was strictly controlled to be identical between morning and afternoon sessions, the heightened reactivity persisted in the morning. This robust pattern strongly implicates endogenous circadian mechanisms—rather than residual sleep effects or the simple timing of awakening—as the primary driver [43,44]. Therefore, the modest phase advance of wake time implemented in our study is unlikely to have substantially confounded the vascular reactivity, as the key driver appears to be the underlying circadian phase at the time of exercise. Furthermore, we have used delta calculations to eliminate the impact of fluctuations in baseline fluctuations in vascular parameters. Future studies should consider stricter control of both sleep quality and timing (e.g., employing advanced tools such as polysomnography for high-precision sleep monitoring) to disentangle the independent effects of exercise timing from sleep-related influences. Lastly, non-invasive measurements restricted the assessment of endothelial cell phenotypes and biochemical changes associated with vascular function. Future research should consider integrating in vitro models or animal studies to delve into the molecular and cellular mechanisms underlying the timing of exercise on vascular health.

## 5. Conclusions

Exercise induces time-dependent hemodynamic responses in healthy sedentary adults. Considering the effects of exercise on reducing blood pressure and vascular resistance, optimizing cerebral blood flow, and improving wall shear stress patterns, aerobic exercise at 06:00 may provide the greatest benefits. Secondly, aerobic exercise at 18:00 also has certain advantages, reflected in increased blood flow and wall shear stress. However, for individuals with cardiovascular diseases, considering that surges in resting blood pressure and immediate post-exercise blood pressure increases may exceed their tolerance, afternoon exercise might be a better choice for them. These findings highlight the critical role of timing in optimizing exercise interventions for vascular health.

## Figures and Tables

**Table 1:** Baseline characteristics of the subjects.

Characteristics	Values
Age (years)	22.05 ± 0.42
Height (cm)	176.50 ± 1.07
Weight (kg)	65.95 ± 1.22
BMI (kg/m^2^)	21.17 ± 0.32
WHR	0.89 ± 0.02
HR_max_ (bpm)	198.00 ± 0.42
HR_rest_ (bpm)	66.18 ± 0.65
HRt__mean_ (bpm)	151.8 ± 0.35

BMI, body mass index; WHR, waist-to-hip ratio; HR_max_, maximum heart rate; HR_rest_, resting heart rate; HRt__mean_, mean of target heart rates.

## Data Availability

All data generated or analyzed during this study are included in this article. More detailed data are available upon request from the corresponding authors.
